# Epigenetic profiling for prognostic stratification and personalized therapy in breast cancer

**DOI:** 10.3389/fimmu.2024.1510829

**Published:** 2025-01-14

**Authors:** Xiao Guo, Chuanbo Feng, Jiaying Xing, Yuyan Cao, Tengda Liu, Wenchuang Yang, Runhong Mu, Tao Wang

**Affiliations:** ^1^ School of Pharmacy, Beihua University, Jilin, China; ^2^ School of Basic Medical Sciences, Beihua University, Jilin, China; ^3^ Research Laboratory Center, Guizhou Provincial People’s Hospital, Guiyang, Guizhou, China

**Keywords:** breast cancer, epigenetics, machine learning, personalized therapy, vincristine

## Abstract

**Background:**

The rising incidence of breast cancer and its heterogeneity necessitate precise tools for predicting patient prognosis and tailoring personalized treatments. Epigenetic changes play a critical role in breast cancer progression and therapy responses, providing a foundation for prognostic model development.

**Methods:**

We developed the Machine Learning-derived Epigenetic Model (MLEM) to identify prognostic epigenetic gene patterns in breast cancer. Using multi-cohort transcriptomic datasets, MLEM was constructed with rigorous machine learning techniques and validated across independent datasets. The model’s performance was further corroborated through immunohistochemical validation on clinical samples.

**Results:**

MLEM effectively stratified breast cancer patients into high- and low-risk groups. Low-MLEM patients exhibited improved prognosis, characterized by enhanced immune cell infiltration and higher responsiveness to immunotherapy. High-MLEM patients showed poorer prognosis but were more responsive to chemotherapy, with vincristine identified as a promising therapeutic option. The model demonstrated robust performance across independent validation datasets.

**Conclusion:**

MLEM is a powerful prognostic tool for predicting breast cancer outcomes and tailoring personalized treatments. By integrating epigenetic insights with machine learning, this model has the potential to improve clinical decision-making and optimize therapeutic strategies for breast cancer patients.

## Introduction

Breast cancer (BC) is currently one of the most prevalent cancers among women, exhibiting significant heterogeneity that necessitates varying treatment approaches at different stages (1). For instance, triple-negative BC can be subdivided into multiple subtypes, each with distinct molecular characteristics and varying sensitivities to different therapeutic agents (2). Despite substantial advancements in understanding the pathogenesis of BC, morbidity and mortality rates, especially post-metastasis, remain high. This underscores the importance of accurate prognosis prediction and personalized treatment strategies for BC patients, which hold promising potential for improving outcomes.

Epigenetics, which involves heritable changes not affecting the DNA sequence, plays a crucial role in tumor development through mechanisms such as DNA methylation, histone modification, chromatin remodeling, and alterations in non-coding RNA (3). Studies have demonstrated the significant relationship between epigenetics and tumor progression. For instance, abnormal DNA methylation modifications can lead to poor prognosis and have been used to construct prognostic models for BC (4). Similarly, histone modifications, like aberrant acetylation, regulate the expression of oncogenes and tumor suppressor genes and are strongly correlated with poor outcomes in BC patients (5). Thus, an in-depth analysis of epigenetic alterations in tumor development could provide new insights and approaches for BC treatment.

Machine learning, a crucial branch of artificial intelligence, offers potential solutions to issues of poor reproducibility in current group learning methodologies (6). Recently, various machine learning algorithms have been employed to develop clinical prediction models, particularly for cancer diagnosis and prognosis. For example, a multi-gene prognostic model for ovarian cancer has proven effective in assessing patients’ conditions and guiding clinical treatment (7). Individualized risk assessments are essential for providing patients with accurate prognostic counseling and tailored clinical treatment plans.

This study presents a groundbreaking predictive model rooted in epigenetics, employing advanced machine learning techniques to improve prognosis evaluations. By incorporating extensive bioinformatics data, this research seeks to overcome the limitations of conventional models and provide deeper insights into the role of epigenetics in cancer progression. This advancement marks a significant step towards creating patient-specific therapeutic strategies.

## Methods

### Data acquisition

We aggregated data from 11 independent breast cancer cohorts derived from four databases: The Cancer Genome Atlas (TCGA), the Gene Expression Omnibus (GEO), and Metabric (8). For a robust and comprehensive prognostic analysis, we focused on samples with complete survival data. Detailed dataset characteristics, including platform usage and cohort breakdowns, are summarized in **Supplementary Table S1**. Epigenetic regulators were sourced from the EpiFactors database (9).

### Machine learning-assisted epigenetic signature

Utilizing the approach of Liu et al. (10), we incorporated 10 computational tools to construct a distinctive telomerase signature for breast cancer: Random Survival Forest (RSF), Least Absolute Shrinkage and Selection Operator (LASSO), Gradient Boosting Machine (GBM), Survival Support Vector Machine (Survival-SVM), Supervised Principal Component (SuperPC), Ridge Regression, Partial Least Squares Cox Regression (plsRcox), CoxBoost, Stepwise Cox Regression, and Elastic Net (Enet). RSF, LASSO, CoxBoost, and Stepwise Cox played crucial roles in dimensionality reduction and variable selection, resulting in 108 unique configurations to create a predictive signature. We assessed all cohorts, including TCGA and eight external datasets, using the average Concordance index (C-index) to determine the most reliable prognostic model, thereby establishing a specific signature for predicting breast cancer outcomes.

To further refine our model and ensure it included only the most predictive genes, we employed an exhaustive search strategy. After identifying 28 candidate genes using RSF and univariate Cox proportional hazards regression, we evaluated all possible combinations of these genes to identify the subset that provided the best model performance. Performance was assessed using predefined criteria, including the C-index and Akaike Information Criterion (AIC). This process reduced the candidate genes to nine, which demonstrated the highest prognostic value across all training datasets.

A risk score for each patient was then calculated using the expression levels of the selected genes weighted by their regression coefficients from the Cox proportional hazards model. The final nine-gene signature was validated across multiple independent cohorts, demonstrating consistent predictive accuracy for breast cancer outcomes.

### Genomic alteration analysis in MLEM groups

We analyzed genetic variations between two MLEM groups using the TCGA-BRCA database, focusing on both mutation levels and Copy Number Alterations (CNA). Tumor Mutation Burden (TMB) for high- and low-MLEM breast cancer patients was derived from raw mutation data, with the most frequently mutated genes (mutation rate > 5%) visualized via maftools. Patient-specific mutational signatures were identified using the deconstructSigs tool (11), revealing four significant mutational signatures (SBS2, SBS13, SBS7b, SBS7d) with elevated mutation frequencies. Additionally, we identified the five most common regions of amplification and deletion, particularly in genes located at 17q23.1 and 15q13.1.

### Single-cell RNA sequencing data processing

To process single-cell RNA sequencing data, we utilized the GSE161529 dataset with Seurat (v4.0) (12). Initially, we excluded genes with zero expression, focusing only on those with detectable expression levels. The expression matrix was normalized using Seurat’s “SCTransform” function, followed by dimensionality reduction via PCA and UMAP techniques. Cellular groupings were identified using the “FindNeighbors” and “FindClusters” functions. The DoubletFinder package was applied to remove potential doublets, ensuring data integrity (13). Cells with over 15% mitochondrial genes or fewer than 500 genes were excluded. Ultimately, 50,214 cells passed quality control and were categorized by manually annotating cell types based on established marker genes.

### Adapting SCENIC for gene regulatory network inference

In our study, we adapted the Single-Cell rEgulatory Network Inference (SCENIC) methodology to construct gene regulatory networks (GRNs) from single-cell RNA sequencing data (14). SCENIC functions through a three-step process: identifying co-expression modules between transcription factors (TFs) and potential targets, pinpointing direct targets using enriched TF motifs, and defining regulons composed of a TF and its direct targets. We calculated the regulatory activity score (RAS) for each cell using the area under the recovery curve. To address SCENIC’s limitations with large datasets and sequencing depth variability, we preprocessed data into metacells, enhancing scalability and robustness, thereby significantly improving data handling and computational efficiency (15).

### Regulon clustering in regulatory crosstalk analysis

Our study employs an advanced computational approach to map the regulatory crosstalk among TFs and their target genes, with a focus on TF clustering. The process starts by filtering TF-target interaction data to concentrate on significant pairs (significance threshold > 1), emphasizing the most relevant regulatory interactions. Key regulatory TFs, termed hub genes, are identified by quantifying their regulation of target genes. These interactions are represented using an undirected graph model, spatially refined by a force-directed algorithm to clearly illustrate the network architecture and TF-target interactions. Additional structural insights are obtained through the Leiden algorithm, which detects community structures and groups TFs into clusters based on their regulatory links, enhancing our understanding of the regulatory landscape.

### Cell-cell communication analysis

We employed the “CellChat” R package to analyze cell-cell communication (16), creating CellChat objects from UMI count matrices and utilizing the “CellChatDB.human” database for ligand-receptor interactions. The analysis was conducted with default settings, merging objects via the “mergeCellChat” function. Differences in interaction number and intensity across cell types were visualized using “netVisual_diffInteraction.” Changes in signaling pathways were assessed with “rankNet,” while gene expression distributions were illustrated using “netVisual_bubble” and “netVisual_aggregate.” Additionally, the NicheNet package was used to explore ligand activity and the regulated expression of downstream targets (17), providing deeper insights into signaling dynamics and communication pathways within the cellular microenvironment.

### Evaluation of TME disparities and immunotherapy response

To evaluate immune cell infiltration in the tumor microenvironment (TME), we employed multiple algorithms: MCPcounter, EPIC, xCell, CIBERSORT, quanTIseq, and TIMER (18–23). These analyses enabled patient categorization by their MLEM scores, offering a detailed view of the immune landscape. To benchmark their consistency, we calculated Spearman’s correlation coefficients between the outputs of these methods for major immune cell populations. We also assessed the ESTIMATE and TIDE indices to understand immunotherapy potential and prognostic implications for breast cancer (24, 25). Quantification of immune checkpoints was conducted to predict patient responsiveness to immune checkpoint inhibitor (ICI) therapy, thereby supporting personalized medicine and optimizing treatment strategies.

### Therapeutic target and drug identification for high-MLEM patients

To identify potential therapies for high-MLEM patients, we first filtered out duplicate compounds from the Drug Repurposing Hub, resulting in 6,125 unique compounds. We performed Spearman correlation analysis to select genes associated with breast cancer outcomes, targeting those with correlation coefficients above 0.15 (P < 0.05) and those indicating poor prognosis with coefficients below -0.15 (P < 0.05). Gene significance was further evaluated using CERES scores from the Cancer Cell Line Encyclopedia (CCLE) in relation to breast cancer cell (26).

We also assessed drug responsiveness using data from the Cancer Therapeutics Response Portal (CTRP) and the PRISM project, which involve drug screening across various cancer cell lines. The predictive accuracy of drug responses was enhanced using the pRRophetic package’s ridge regression model, validated through 10-fold cross-validation (27).

Furthermore, we explored potential drugs using Connectivity Map (CMap) analysis by comparing gene expression profiles and identifying compounds inversely related to CMap scores, indicating higher therapeutic potential against breast cancer.

### Patient stratification in breast cancer research

For gene expression analysis in breast cancer samples, RNA was extracted using TRIzol reagent (Invitrogen, Carlsbad, CA, USA), and cDNA synthesis was performed using GoScript reverse transcriptase and Master Mix (Promega) according to the manufacturer’s instructions. Quantitative expression was measured via qRT-PCR on the CFX96 Touch Real-Time PCR Detection System (BioRad, Hercules, CA, USA) utilizing the 2^-ΔΔCq^ method with GAPDH as a normalization control. Patients were then stratified based on gene expression levels calculated using a formula derived from the MLEM, aiding in the identification of varying risk profiles and the development of tailored treatment strategies.

### Immunohistochemistry analysis

Breast cancer tissue samples were collected from 30 patients at Guizhou Provincial People’s Hospital, and Hematoxylin and Eosin (HE) staining was performed following standard protocols, with diagnoses confirmed by two independent pathologists. For immunohistochemistry (IHC) analysis on paraffin-embedded samples, procedures and scoring systems from our previous studies were followed (28, 29). Protein expression levels were independently assessed by the same pathologists, ensuring consistency with our earlier research (29).

## Results

### Construction of epigenetic model based on machine learning

**Figure 1:**
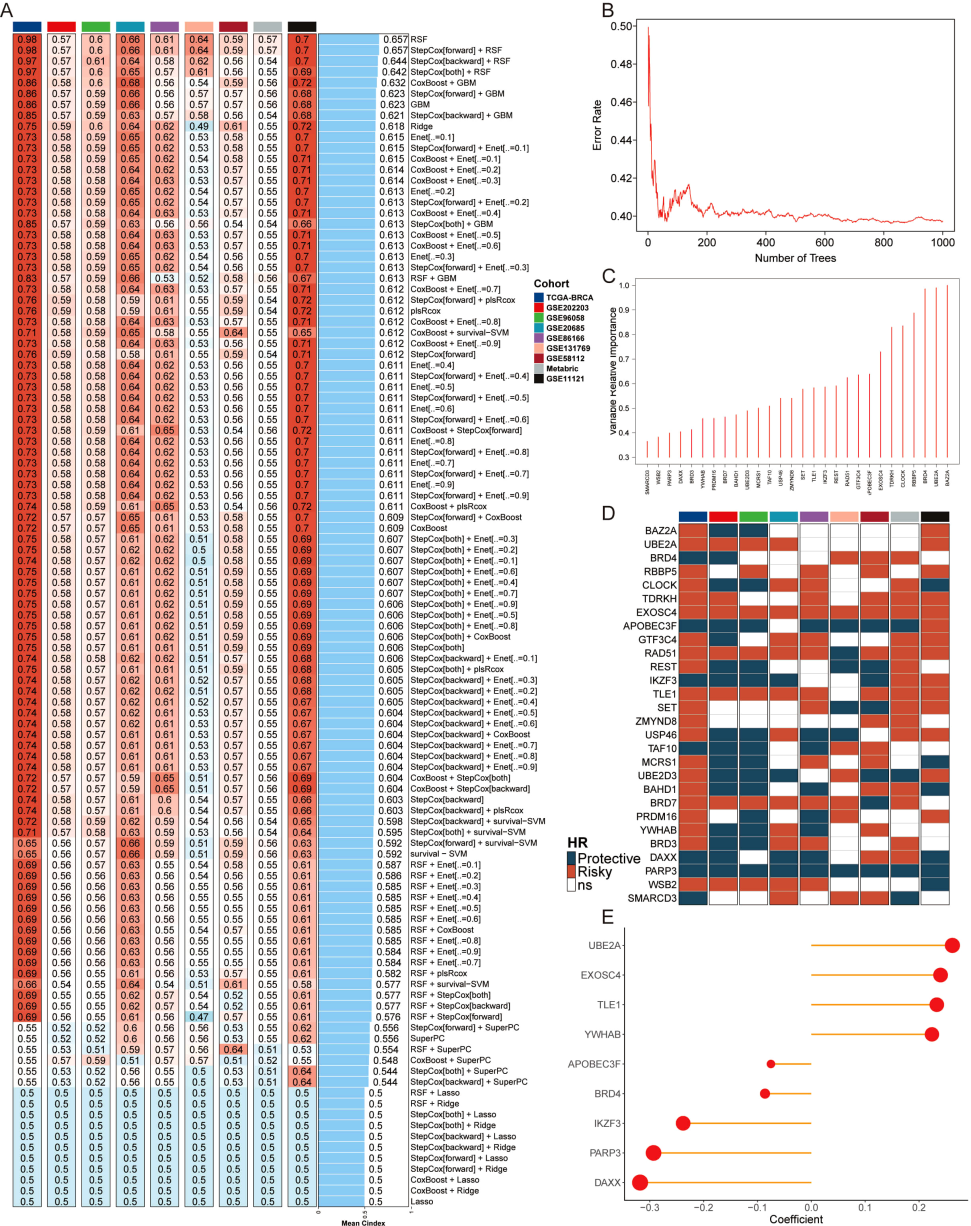
Construction of epigenetic model based on AI. **(A)** Average C-index of 108 combination algorithms in nine BC cohorts. **(B)** Error rate of the RSF in 1000 iterations. **(C)** Importance of 28 epigenetic genes. **(D)** Kaplan-Meier survival analysis of 28 candidate genes across 9 BC cohorts, illustrating their association with patient outcomes. Each gene’s hazard ratio and p-value are shown, highlighting their prognostic significance. **(E)** Correlation coefficients of key genes used in model.

To assess the correlation between epigenetic regulation and BC prognosis, we collected epigenetic regulators from the EpiFactors database and applied them across nine independent cohorts. This multi-platform dataset enabled the construction of a robust model using machine learning techniques. The model’s performance was evaluated by calculating the average C-index across the nine cohorts using 10 machine learning algorithms in 108 combinations (**Figure 1A**). The RSF model, which achieved the highest score, was selected as the final model. Genes corresponding to the point with the lowest error rate were chosen as candidate variables (**Figures 1B, C**). Subsequently, a univariate Cox analysis was performed on these genes to assess their impact on BC prognosis (**Figure 1D**). Ultimately, nine genes were identified to construct a machine learning-based epigenetic model (MLEM) (**Figure 1E**). The MLEM effectively stratified BC patients into different risk groups with accurate prognostic predictions (**Supplementary Figure S1**).

### Evaluation of MLEM with 68 published BC models

Univariate and multivariate Cox analyses were utilized to compare MLEM with other clinical indicators, revealing that MLEM possessed strong predictive capability (**Supplementary Figure S2A**). A nomogram incorporating MLEM, staging, and age was developed to predict the overall survival (OS) of patients at 1, 3, and 5 years (**Supplementary Figure S2B**). The analysis of calibration curves and Hosmer-Lemeshow tests showed a high level of consistency with the standard curve (**Supplementary Figures S2C, D**). Additionally, decision curve analysis (DCA) suggested that MLEM holds significant potential for clinical application (**Supplementary Figure S2E**). Compared to other clinical factors, MLEM demonstrated superior discrimination (**Supplementary Figure S2F**).

**Figure 2:**
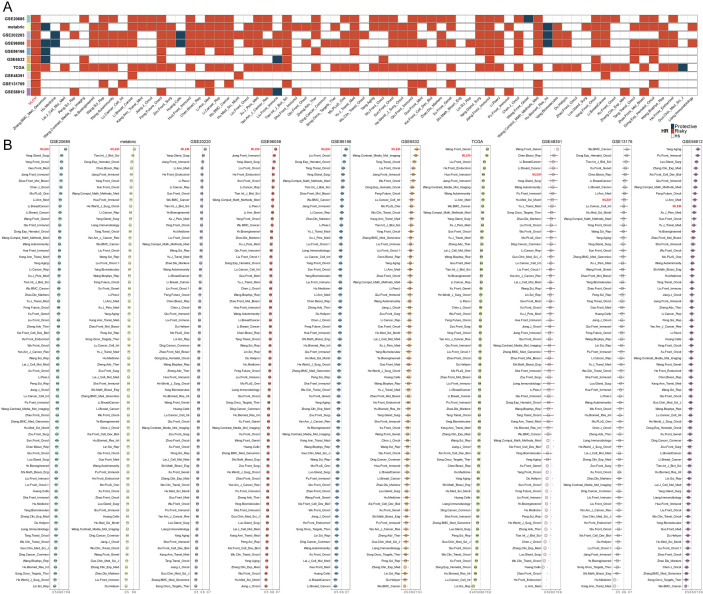
Evaluation of MLEM with 68 published BC models. **(A)** univariate Cox analysis of models in 10 BC cohorts. **(B)** Comparison of the average C-index of models in 10 BC cohorts.

Subsequently, we manually curated 68 published BC models for evaluation against MLEM. Univariate Cox analysis was conducted on these models across 10 BC cohorts. MLEM exhibited the best stability across all 10 datasets (**Figure 2A**). Furthermore, the predictive ability was evaluated using the average C-index, where MLEM consistently ranked high in all 10 cohorts, underscoring its robustness and effectiveness in BC prognostication (**Figure 2B**).

### Multi-omics analysis of genome variations for MLEM

**Figure 3:**
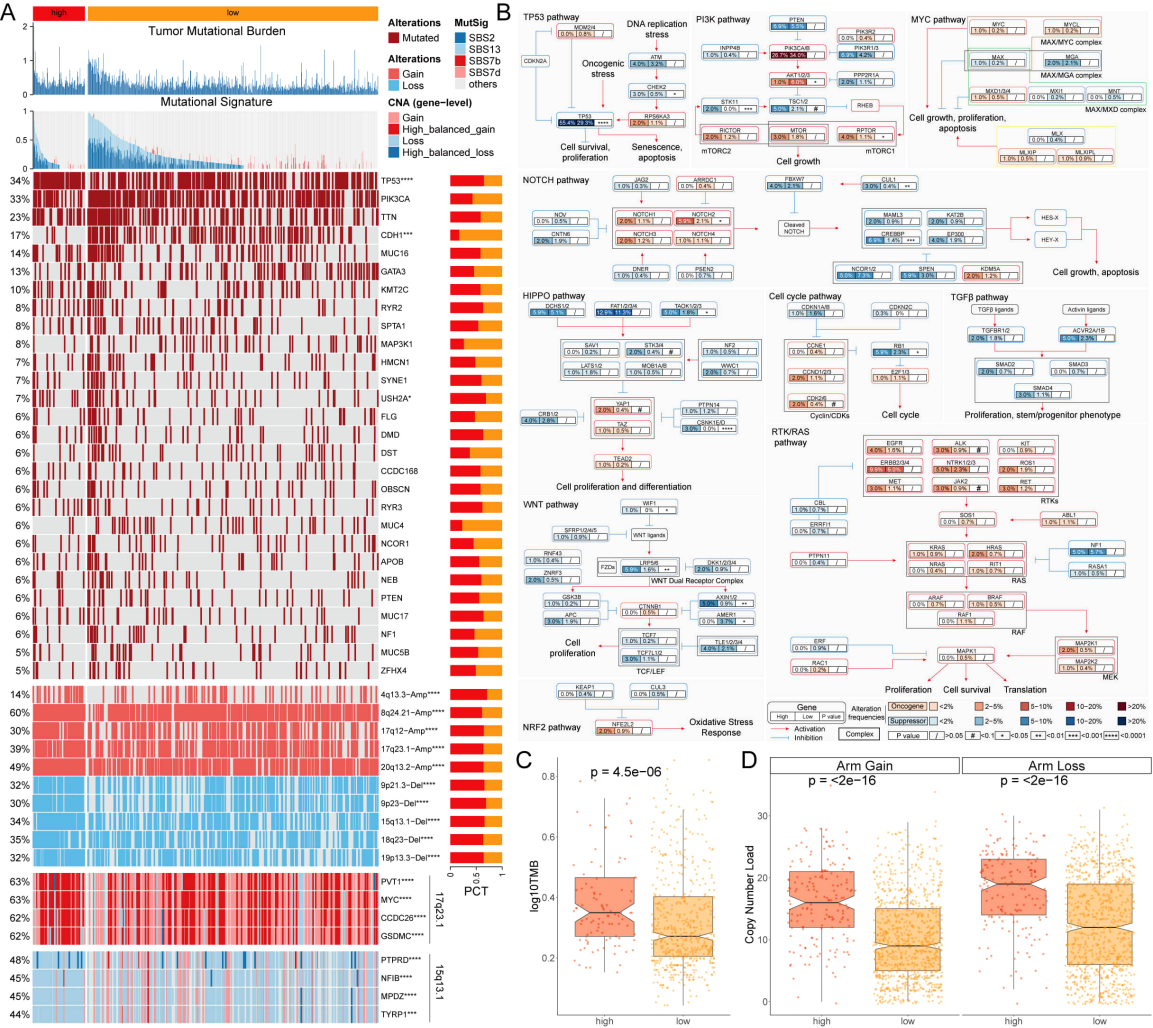
Multi-omics analysis of genome variations for MLEM. **(A)** Genomic alteration landscape of MLEM, from up to bottom: TMB, gene mutational signatures, gene mutation frequency, CNAs (the red represents amplification, and the blue represents deletion), and the representative genes in region 17q23.1 and 15q13.1. **(B)** Mutation frequency of 10 oncogenic pathways between MLEM groups. **(C)** Comparison of TMB between MLEM groups. **(D)** Amplification or deletion of chromosomal arm. *p < 0.05, ***p < 0.001, ****p < 0.0001.

A multi-omics analysis was conducted on genes potentially associated with the BC process in patients with varying MLEM. The findings revealed that patients with high-MLEM group exhibited a significantly higher tumor mutation burden (TMB) compared to those with low-MLEM, which was accompanied by a higher rate of multigene mutations (**Figures 3A, C**). When integrating mutation data from ten oncogenic signaling pathways, it was observed that the mutation rates of classic tumor suppressor genes such as TP53, STK11, and AXIN1/2 were significantly elevated in the high-MLEM group. Conversely, the mutation rates of classic oncogenic genes such as PIK3CA/B and AKT1/2/3 were lower in the high-MLEM group (**Figures 3A, B**). Additionally, a comparison of copy number variations between the two MLEM groups indicated more pronounced amplifications and deletions of chromosome arms in the high-MLEM group. Notable amplifications included 4q13.3, 8q24.21, 17q12, 17q23.1, and 20q13.2, while significant deletions were observed at 9p21.3, 9p23, 15q13.1, 18q23, and 19p13.3 (**Figures 3A, D**). High-MLEM patients exhibited a greater prevalence of SBS13, which is associated with increased tumor mutation burden and aggressive tumor phenotypes. This finding aligns with the poorer prognosis observed in this group. SBS2 and SBS13, indicative of APOBEC activity, have been shown to correlate with heightened sensitivity to immune checkpoint inhibitors. This may explain the differential therapy responses between MLEM groups. Additionally, the presence of SBS7b and SBS7d suggests potential vulnerabilities to therapies targeting nucleotide excision repair pathways.

### Analysis of biological mechanisms of MLEM at the single-cell level

**Figure 4:**
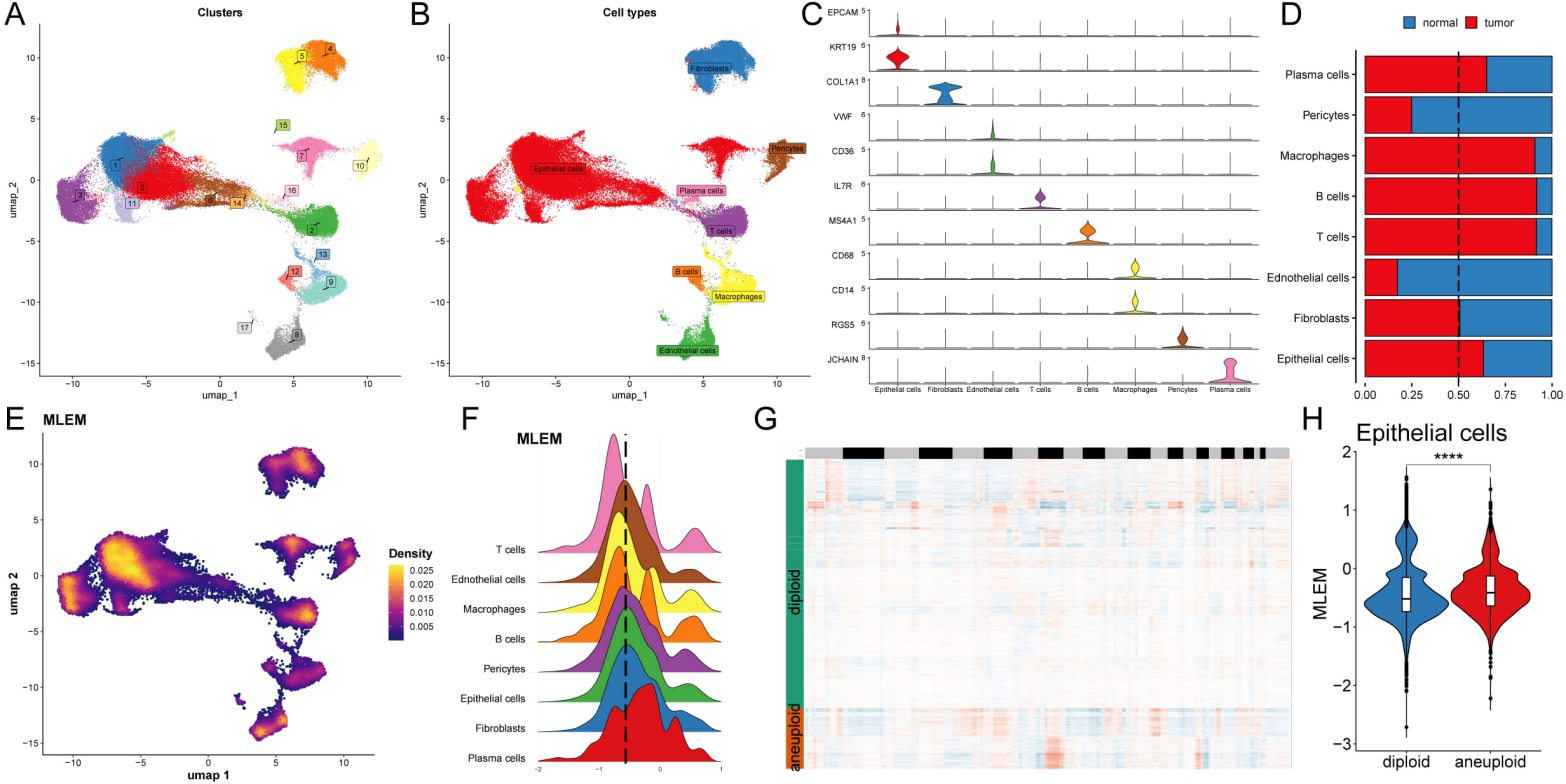
Analysis of biological mechanisms of MLEM at the single-cell level. **(A)** UMAP visuali-zation illustrates the distribution of cell clusters. **(B)** UMAP visualization illustrates the distribu-tion of identified cell types. **(C)** Representative markers of each cell type. **(D)** Proportion of eight cell types of between tumor and normal tissues. **(E)** UMAP visualization illustrates the distribution of MELM value. **(F)** Distribution of MLEM value across various cell types. **(G)** Estimation of copy number using copyKAT algorithm. **(H)** MLEM variance between diploid and aneuploid cells in the epithelial cell. ****p < 0.0001.

Single-cell transcriptome analysis was conducted to evaluate the biological signifi-cance of MLEM in 14 BC patients, comprising 6 normal tissues and 8 BC tissues (**Supplementary Figures S3A, B**). Initially, 18 clusters were identified, and 8 cell types were further determined (**Figures 4A, B**). The proportions of these 8 cell types were then analyzed, highlighting the differences between the patient’s body and the 14 tissue samples (**Supplementary Figures S3C, D**). Veri-fication of these cell types using representative markers confirmed consistency with the original results (**Figure 4C**; **Supplementary Figure S3E**). Analysis of the distribution of these cell types revealed that epithelial cells, plasma cells, macrophages, T cells, and B cells were more prevalent in BC tissue, whereas pericytes and endothelial cells were more abundant in normal tissue. Fibroblasts were found in similar proportions in both BC and normal tissues (**Figure 4D**).

The previously established MLEM was integrated into the single-cell analysis and categorized into high- and low-MLEM groups based on the peak of epithelial cells (**Figures 4E, F**). Differential gene expression between the MLEM groups was then analyzed across these 8 cell types (**Supplementary Figure S3F**). For instance, in epithelial tumor cells, genes related to rRNA binding, translation regulatory factors, and nucleic acid binding were significantly upregulated in high-MLEM cells, whereas genes associated with histone binding were significantly upregulated in low-MLEM cells (**Supplementary Figure S3G**). Additionally, the copy number alteration of genes in epithelial cells was examined using the CNA-based copyKat algorithm (**Figure 4G**), revealing that aneuploid tumor cells were more prevalent than diploid normal cells (**Figure 4H**).

### Exploring specific regulatory factors for MLEM

**Figure 5:**
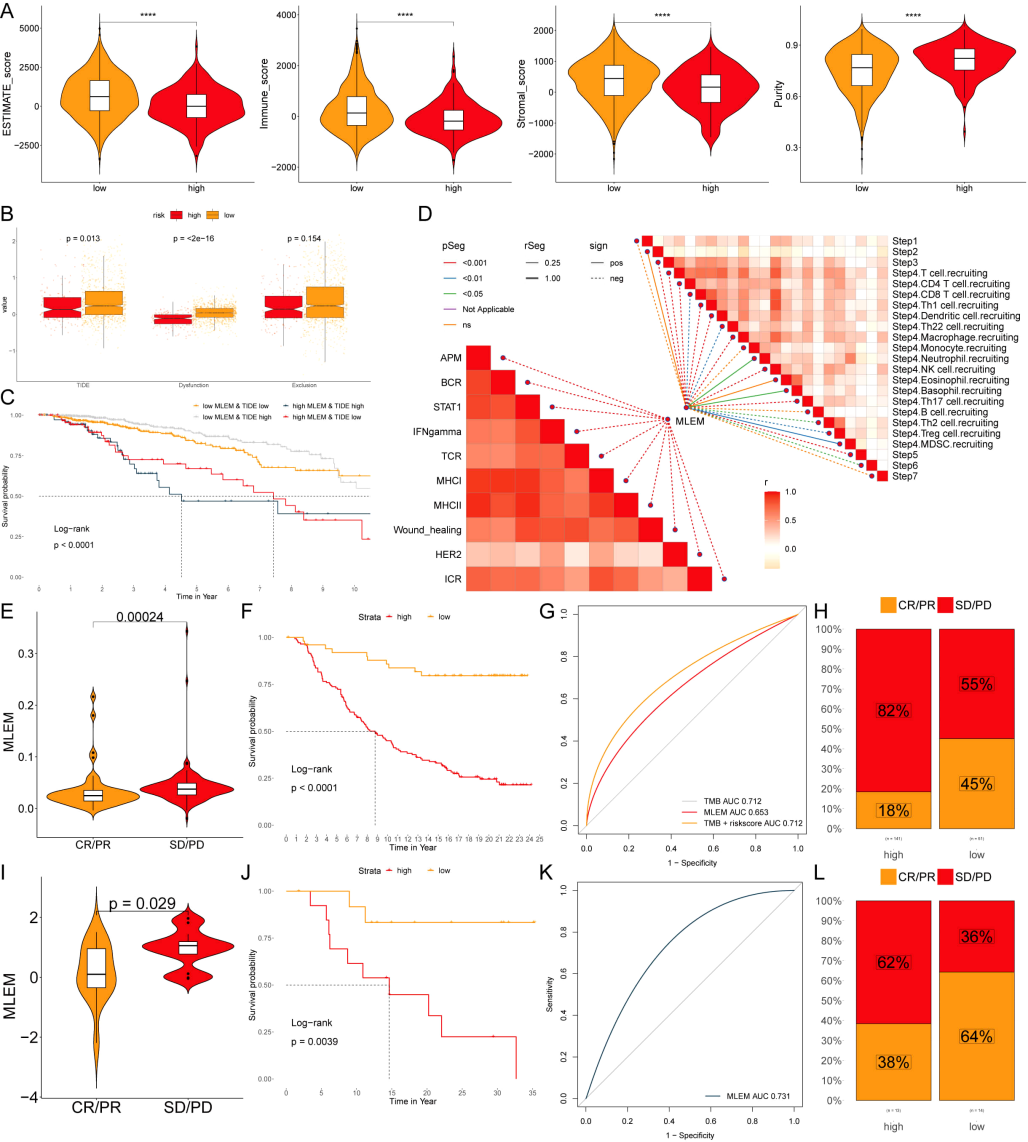
Exploring specific regulatory factors for MLEM. **(A)** umapRAS visualization illustrates the distribution of cell clusters. **(B)** umapRAS visualization illustrates the distribution of identified cell types. **(C)** Variance analysis plot highlights the PC1 impact of cell types. **(D)** Variance analysis plot highlights the PC2 impact of MLEM. **(E)** Regulons ranking for each cell type based on RSS. **(F)** Three top regulons focus on epithelial cells. **(G)** Interactions network of regulons constructed using the Leiden algorithm. **(H)** Detail network of modules A and C. **(I)** Functional variations linked to MLEM in epithelial cells. **(J)** Representative pathways activated or inhibited in the context of high-MLEM. **(K)** TFs involved in translation initiation. **(L)** Detailed regulatory network of the interactions among TFs involved in translation initiation. ****p < 0.0001.

To comprehensively construct GRNs, we utilized the SCENIC pipeline to analyze single-cell RNA-seq data along with cis-regulatory sequence information. This process transformed the gene expression data into RAS for TFs (**Figures 5A, B**). We then performed variance decomposition analysis using principal component analysis (PCA) to identify specific regulons for MLEM and different cell types. The results indicated that PC1 accounted for cell type-specific TFs, while PC2 was associated with MLEM-specific TFs (**Figures 5C, D**).

In our investigation to identify key regulators of cell identity, we evaluated the activity of each regulon across various cell types. We assigned a regulon specificity score (RSS) based on Jensen-Shannon divergence to measure each regulon’s association with specific cell identities. By concentrating on regulons with the highest RSS values, we analyzed their functional characteristics (**Figure 5E**). This analysis identified MESP1, SPDEF, and GATA3 as key regulons uniquely associated with epithelial cells. UMAP plots further highlighted the specificity of these regulons’ activities within epithelial cells (**Figure 5F**). Additionally, we presented and analyzed regulons specific to other cell types, underscoring the distinct regulatory networks that define cell identities (**Supplementary Figure S4A**).

Recognizing that TFs collaborate to regulate gene expression, we systematically examined the combinatory patterns of these regulatory elements. By assessing the similarity of RAS scores for each regulon pair across the entire atlas using the Leiden algorithm, we organized 343 regulons into 12 distinct modules, unveiling complex regulatory patterns within the cellular landscape (**Figure 5G**; **Supplementary Figure S4B**). Notably, modules A and C were predominantly associated with MLEM (**Figure 5H**). We then investigated the specific TFs driving transcriptomic changes in epithelial cells influenced by MLEM. GSEA identified several pathway alterations (**Figure 5I**), including the translation initiation in epithelial cells with high-MLEM (**Figures 5I, J**). Further analysis identified the TFs contributing to this pathway and their roles in MLEM progression (**Figure 5K**). A comprehensive regulatory network is illustrated in **Figure 5L**.

### Intercellular communication between MLEM groups

**Figure 6:**
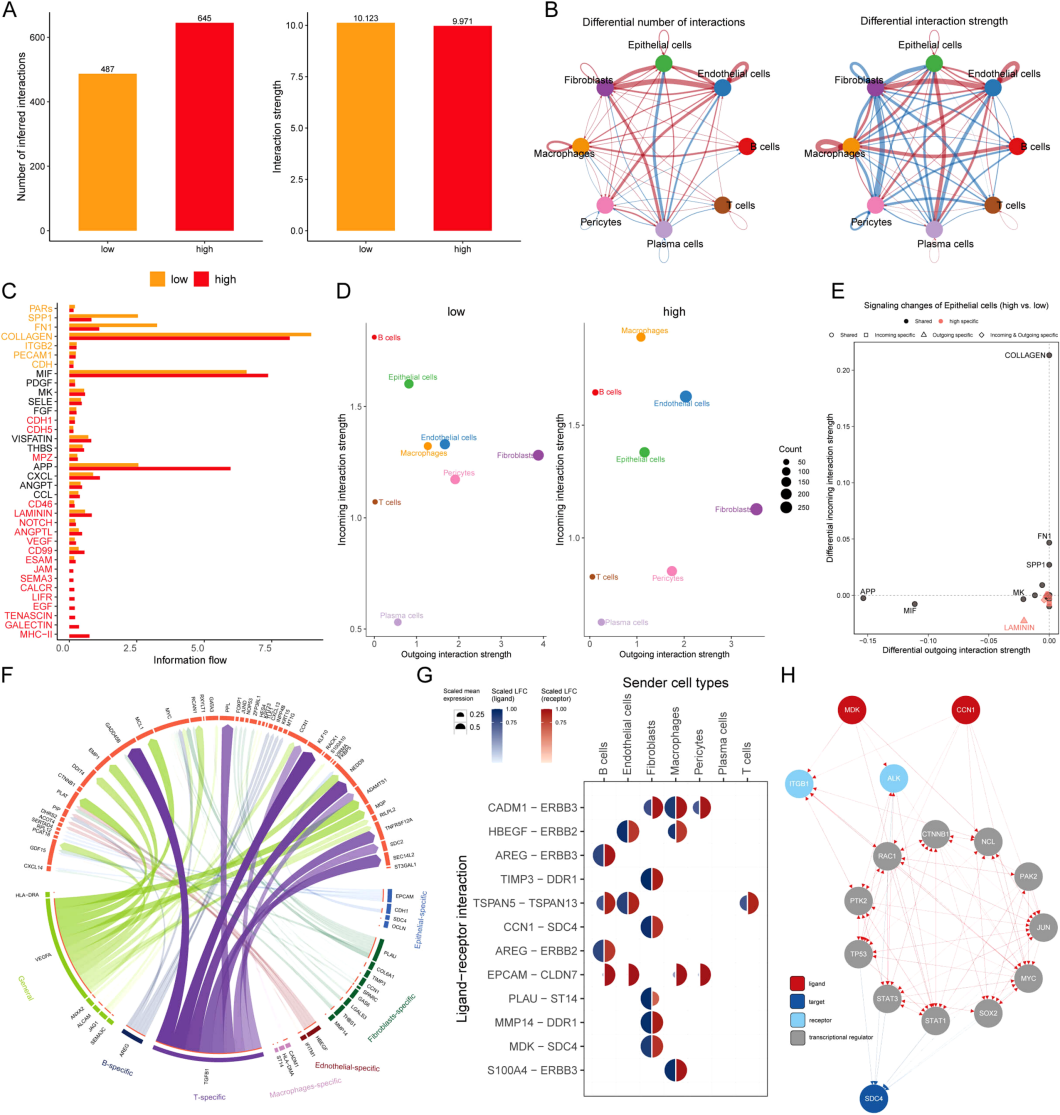
Intercellular communication between MLEM groups. **(A)** Comparison of the number and intensity of intercellular communication between MLEM groups. **(B)** Network diagrams illustrate varied interaction strengths among each cell type. **(C)** Active communication pathways in MLEM groups. **(D)** Scatter plots compare outgoing and incoming interaction strengths between cell types. **(E)** Pathway specificity in epithelial cells within high-MLEM. **(F)** Potential ligand-receptor interactions, inferred through NicheNet analysis. **(G)** Circos plot summarizes top-predicted ligand-receptor pairs in high MLEM cells. **(H)** The routes of MDK and CCN1 ligands to the target receptor SDC4.

The relationships of intercellular communication differences between MLEM groups among 8 types of cells were analyzed using CellChat. Although the type of cellular communication was more diverse, the intensity of interactions was slightly lower in high-MLEM cells (**Figure 6A**). Differences in intercellular communication revealed a significant decrease in the number of interactions involving plasma cells with other cells, whereas interactions among the other cells significantly increased in high-MLEM cells (**Figure 6B**). Additionally, the analysis of intercellular signaling pathways showed that seven pathways were highly active in low-MLEM cells, while 18 pathways were highly active in high-MLEM cells (**Figure 6C**).

Further analysis assessed the strengths of outgoing and incoming interactions to gauge cell-cell communication. Compared to low-MLEM cells, macrophages and endothelial cells showed a significant increase in their signal reception ability, whereas plasma cells and T cells exhibited a significant decrease in high-MLEM cells (**Figure 6D**). Several pathways in epithelial cells were specific to MLEM, such as LAMININ (**Figure 6E**).

A deeper analysis was performed to summarize the expression of receptor ligands in the most valuable signaling pathways (**Figure 6F**). It was found that MDK-SDC4 and CCN1-SDC4 ligand-receptor pairs were highly expressed in fibroblasts (**Figure 6G**). Finally, signaling communication pathways involving MDK-SDC4 and CCN1-SDC4 ligand-receptors were analyzed, identifying potential pathways (**Figure 6H**).

### MLEM predicts immunotherapy response

**Figure 7:**
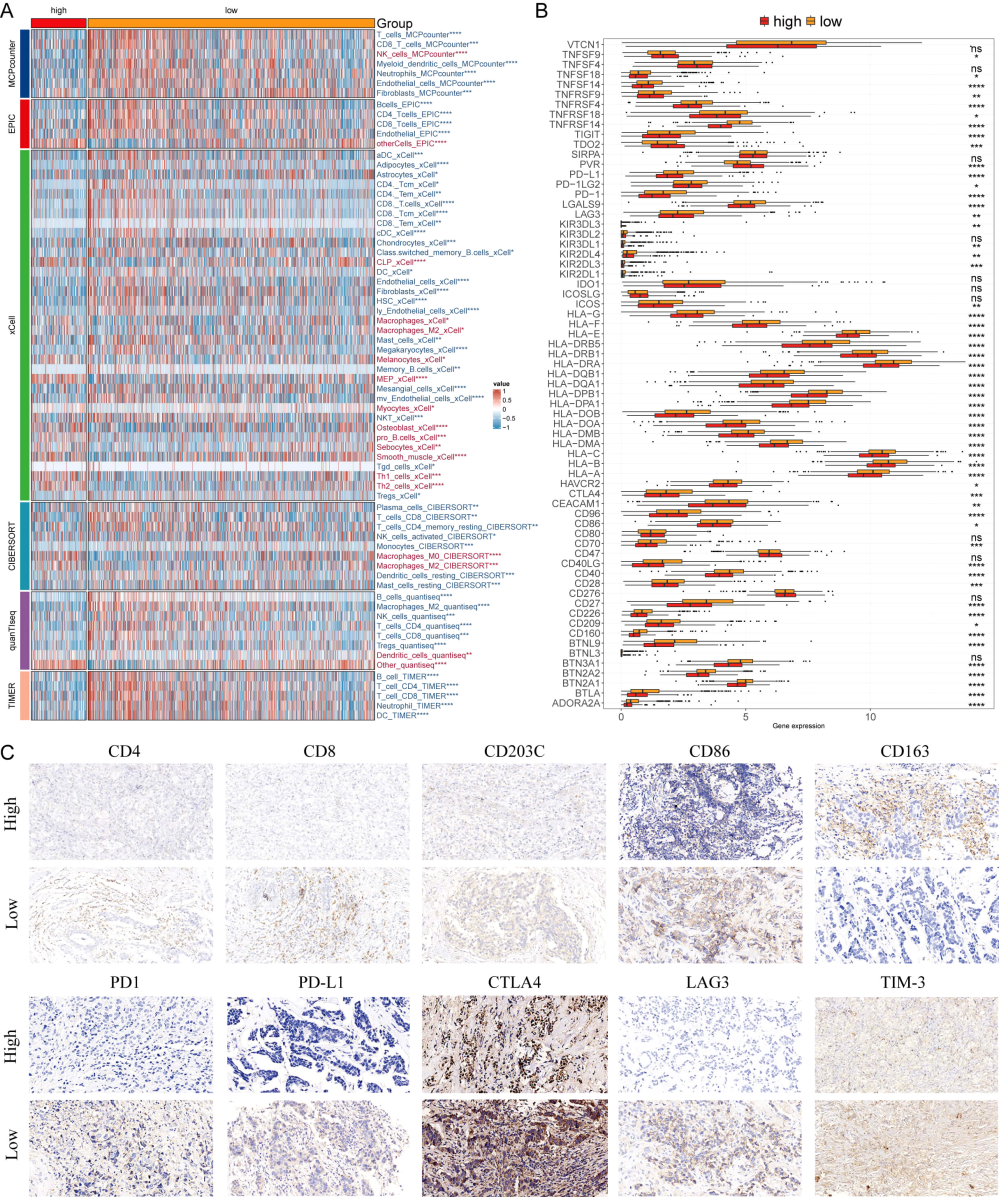
Differential expression and immunohistochemical analysis of immune markers in tumor microenvironments between MLEM subgroups. **(A)** Heatmap provides a comparative view of immune cell infiltration in tumor samples with low and high-MLEM, utilizing various computational algorithms for quantification. Each row represents a different type of immune cell, with the color intensity reflecting the level of infiltration. **(B)** Box plots illustrate the distribution of gene expression levels for immune checkpoint inhibitors (ICIs) across low vs. high MLEM conditions, with statistical significance denoted by ns for not significant; *P < 0.05; **P < 0.01; ***P < 0.001; ****P < 0.0001. **(C)** Representative immunohistochemistry images showcase the staining intensity of various immune markers between high and low expression conditions, visually depicting the differential expression of these markers in correlation with MLEM levels.

Six algorithms were utilized to evaluate the immune microenvironment, revealing that patients in the low-MLEM group exhibited a higher degree of immune cell infiltration, including CD4^+^ and CD8^+^ T cells (**Figure 7A**). The comparison of immune checkpoint inhibitors (ICIs) indicated higher expression levels of ICIs in the low-MLEM group, further suggesting that immunotherapy might be more suitable for these patients (**Figure 7B**). Representative cell markers were evaluated using IHC experiments (**Figure 7C**). Spearman’s correlation analysis revealed a high degree of concordance between MCPcounter, quanTIseq, and TIMER for key immune populations such as CD8^+^ T cells (ρ > 0.75). In contrast, xCell exhibited lower concordance with other methods, particularly for macrophages and B cells (ρ < 0.50). Despite these differences, all methods consistently showed higher immune infiltration levels in the low-MLEM group compared to the high-MLEM group. This robustness across algorithms supports the reliability of our findings.

**Figure 8:**
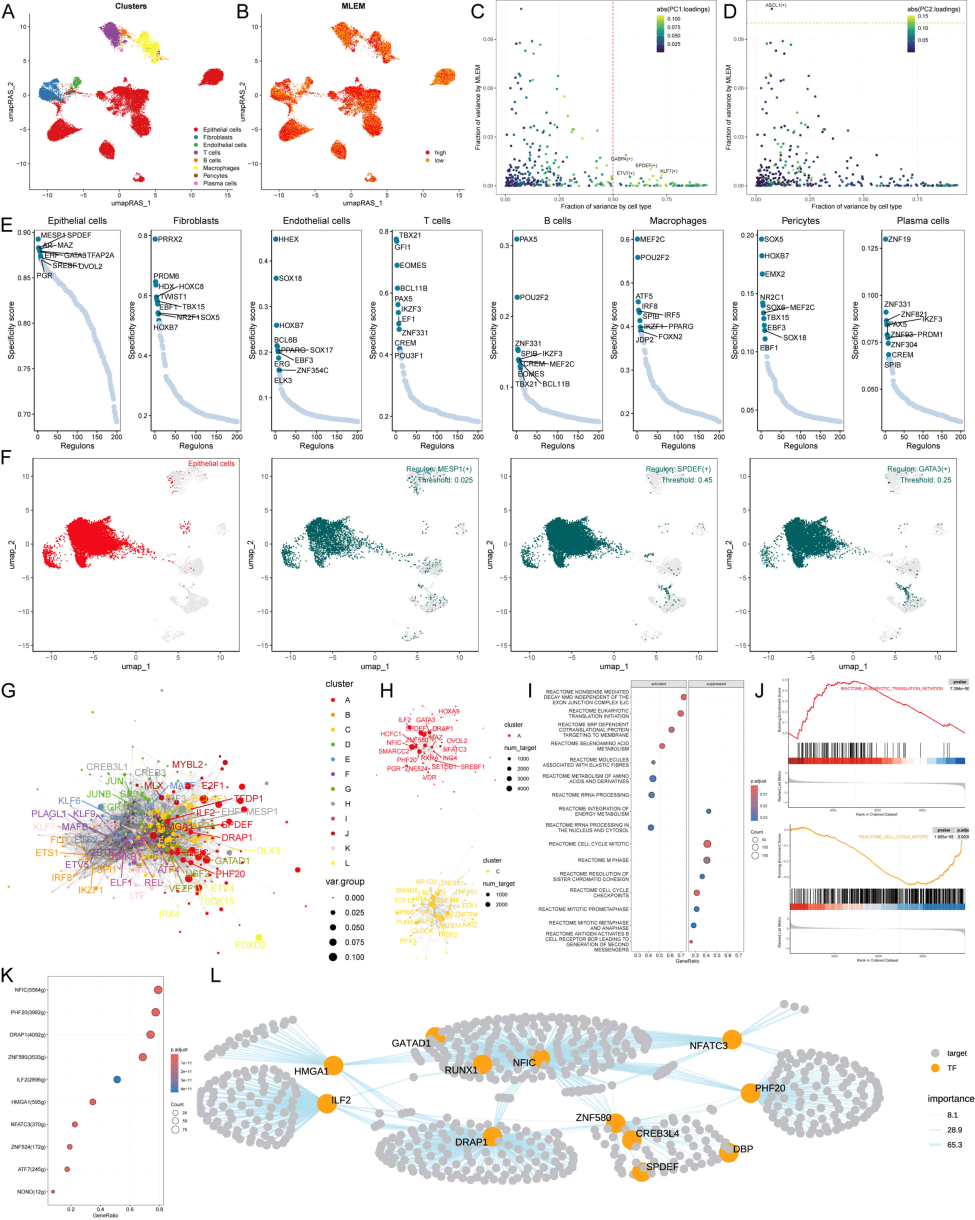
MLEM predicts immunotherapy response. **(A)** ESTIMATE scores, immune scores, stromal scores, and tumor purity between MLEM groups. **(B)** TIDE, dysfunction, and exclusion variations between MLEM groups. **(C)** Survival probability of patients based on the combination of MLEM and TIDE. **(D)** Correlation analysis of MLEM with immune pathways and tumor immune cycle. **(E, I)** Violin charts display the relationship between MLEM levels and responses to anti-PDL1 **(E)** and anti-PD1 **(I)** therapies, detailing the differential immune responses. **(F, J)** Survival probabilities of low and high MLEM patients in anti-PDL1 **(F)** and anti-PD1 **(J)** cohorts, respectively, illustrating the impact of MLEM on survival outcomes. **(G, K)** Analysis estimates the predictive ability of MLEM via AUC values, considering TMB combinations, in anti-PDL1 **(G)** and anti-PD1 **(K)** cohorts, evaluating the efficacy of MLEM as a biomarker. **(H, L)** The percentages of complete response/partial response (CR/PR) and stable disease/progressive disease (SD/PD) in anti-PDL1 **(H)** and anti-PD1 **(L)** cohorts are shown, based on MLEM levels, to assess treatment effectiveness.

Furthermore, patients in the low-MLEM group had superior ESTIMATE scores, immune scores, and stromal scores, whereas high-MLEM patients had higher tumor purity, suggesting that the low-MLEM group might be more responsive to immunotherapy (**Figure 8A**). Subsequently, TIDE analysis showed that TIDE, Dysfunction, and Exclusion indicators were higher in the low-MLEM group compared to the high-MLEM group (**Figure 8B**), and patients in the low-MLEM and low-TIDE group demonstrated significantly better outcomes (**Figure 8C**). Further analysis revealed a significant correlation between immune signaling pathways, the immune cycle, and MLEM in BC (**Figure 8D**). Finally, studies from IMvigor210 (**Figures 8E–H**) and GSE78220 (**Figures 8I–L**) indicated that patients in the low-MLEM group benefited more from PD-L1 and PD-1 administration, confirming that immunotherapy was more suitable for patients in the low-MLEM group.

### Screening drugs for high-MLEM BC patients

**Figure 9:**
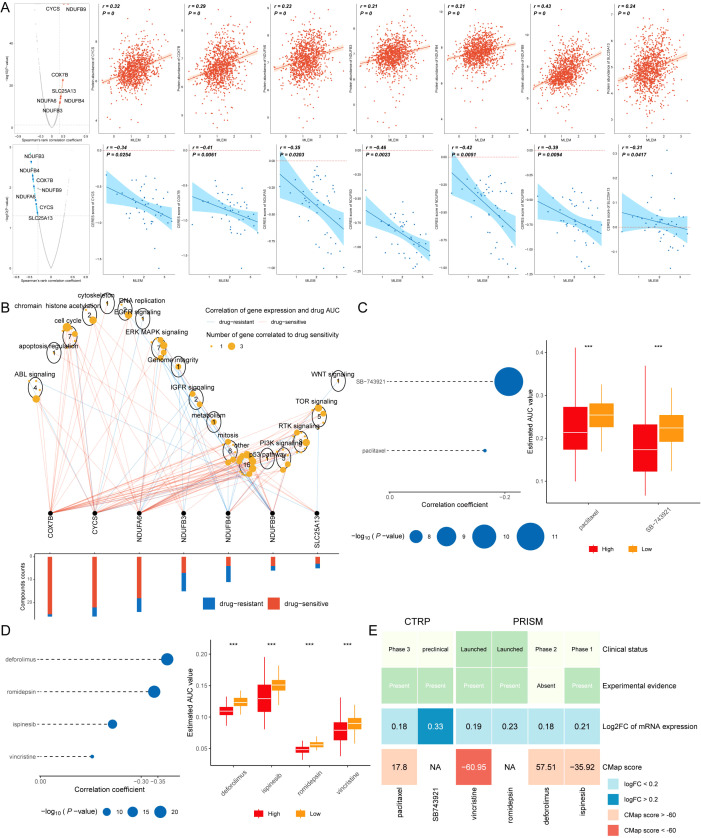
Screening drugs for high-MLEM BC patients. **(A)** Spearman’s correlation illustrating the association between MLEM and the abundance of potential therapeutic targets in breast cancer patients. **(B)** Network analysis highlights the intricate connections between these therapeutic targets and their associated drug action pathways. **(C)** Box plots compare the AUC values of two compounds in the CTRP dataset. **(D)** Box plots compare the AUC values of four compounds in the PRISM dataset. **(E)** Summary table outlines the multi-perspective analysis of the six candidate compounds, detailing their clinical status, experimental evidence, mRNA expression levels, and CMap scores. ***p < 0.001.

Spearman correlation analysis was applied to investigate the correlation between MLEM and gene expression (positive correlation) as well as CERES (negative correlation). This analysis identified seven potential therapeutic targets for patients with high MLEM: CYCS, SLC25AB, COX7B, NDUFB3, NDUFA4, NDUFB6, and NDUFB9 (**Figure 9A**). These targets were found to be closely related to various pathways of action of breast cancer-related drugs, marking them as key therapeutic targets for BC patients (**Figure 9B**).

Subsequently, six potential compounds were identified from the CTPR (SB-743921, paclitaxel) and PRISM datasets (deforlimus, romidepsin, ispinesib, vincristine). The AUC values for these compounds were compared between the two MLEM groups, revealing lower AUC values in high-MLEM patients, which suggests a better chemotherapy efficacy for these patients (**Figures 9C, D**).

A multi-perspective analysis was performed to identify the most effective therapeutic drugs among the six candidates. This analysis incorporated clinical status, experimental evidence, mRNA expression levels, and CMap scores for each compound. Vincristine emerged as the potential therapeutic drug for high-MLEM patients, based on its CMap score (**Figure 9E**).

## Discussion

The mortality of BC is increasing year by year, and the main factor leading to it is the poor prognosis. Different disease courses can greatly affect the accuracy of medication in BC patients, and experiments have demonstrated that BC patients have different treatment options at different stages (2, 3). Epigenetics plays a role by regulating the expression of DNA methylation, and abnormal methylation can have varying degrees of impact on tumor progression, thereby inducing or inhibiting cancer (7). But now there is a lack of models related to BC and epigenetics. Therefore, a prognostic model for BC was established by using 9 epigenetic genes and its mechanism in combination with biological changes in the human body was further elucidated in this study.

The key epigenetic genes were identified using RSF algorithm.We then constructed the epigenetic model based nine genes identified through RSF and Cox proportional hazards regression analyses. These genes were selected for their prognostic significance across multiple cohorts, with high hazard ratios indicating strong associations with breast cancer outcomes. The nine genes were integrated into the MLEM by assigning weights to their expression levels based on regression coefficients, forming a composite risk score for patient stratification. UBE2A has been reported to be associated with the proliferation of ovarian cancer, but the mechanism of action is not yet clear (30), EXOSC4 has been demonstrated to have a carcinogenic effect in diseases such as colorectal cancer, gastric cancer, and lung cancer (31), and its activation can affect the angiogenesis of tumor microenvironment to promote the development of cancers (32), and EXOSC4 can cause cancer by the abnormal methylation of promoter genes (33), improve the prognosis of patients by inhibiting its gene expression and be used to construct prognostic signatures for ovarian cancer (34). Moreover, PARP3, have been found to be associated with BC and can exhibit anti-cancer effects by inhibiting Akt dephosphorylation (35). Some studies have shown that after activation, PAPR3 can improve cancer prognosis by participating in chromosome rearrangement and double-stranded broken strand repair (36). DAXX has been proved to play an anti-tumor role in pancreatic cancer by inhibiting the expression of some genes (37), and improving the repeated sequence and repairing DNA *in vivo* (38).

It was found through the analysis of the mutation landscape of MLEM that the TMB level was higher, the mutation characteristics were more diverse, the gene mutation frequency was higher, and the amplification and deletion of chromosome region were more frequent in high-MLEM group, suggesting the poor prognosis of these patients. Meanwhile, our analysis found that PVT1, MYC, GSDMC, PTPRD, and MPDZ genes were related to the progression of BC. Some studies have shown that PVT1 can interfere with the proliferation and spreading of tumor cells and the angiogenesis in tumor tissues by inhibiting miRNA (39). In the BC process, PVT1 can regulate the expression of some genes by binding to miR-145-5p, resulting in a poor prognosis (40). MYC, as a typical tumor suppressor gene, has a wide range of gene expression regulation capabilities, and is currently used as a target for cancer treatment (41). CCDC26 can regulate miRNA through multiple pathways in thyroid cancer to promote the cancer occurrence (42). GSDMC is also used in the establishment of prognostic models for pancreatic cancer at this stage, indicating that it may be associated with the poor prognosis of pancreatic cancer (43). In summary, the amplification of the above genes suggests that the poor prognosis of BC may be related to it, and imply potential hidden targets for the treatment of BC. PTPRD can inhibit liver cancer by promoting the methylation of DNA in it (44). In addition, MPDZ has also been reported to exert an inhibitory effect on lung cancer by inducing the dephosphorylation of YAP, and its specific expression can also serve as an independent predictive factor for the diagnosis of lung cancer and other cancers (45). In summary, the amplification and deletion of the above genes in the chromosome arm provide a more reasonable explanation for MLEM in evaluating the prognosis of BC patients.

The identification of mutational signatures, such as SBS2, SBS13, SBS7b, and SBS7d, provides insight into the biological underpinnings of breast cancer heterogeneity. These signatures not only influence prognosis but also offer potential therapeutic opportunities. For instance, the APOBEC-related signatures (SBS2, SBS13) may predict responsiveness to immune-based therapies, while UV-related signatures (SBS7b, SBS7d) highlight vulnerabilities to DNA repair-targeting agents.

The tumor immune microenvironment (TIME) includes tumor cells, immune cells, cytokines, etc. These components interact with each other to promote the proliferation of tumors and inhibit tumors, which can also provide an opportunity for tumor escape and are also used in the treatment of tumors now (46). In this study, the levels of immune cell infiltration and ICIs expression in different groups were evaluated. The consistency of immune cell population estimates across different algorithms provides confidence in the robustness of our findings. Notably, MCPcounter, quanTIseq, and TIMER showed strong agreement in estimating CD8^+^ T cell infiltration, aligning with our conclusion that low-MLEM patients exhibit higher immune activity. However, discrepancies observed with xCell, particularly for macrophages and B cells, may stem from differences in algorithmic assumptions (e.g., expression deconvolution versus signature matching). These variations highlight the importance of using multiple approaches to ensure comprehensive immune profiling. Future studies may benefit from experimental validation to complement computational predictions. In low-MLEM group, a significant immune cell infiltration was observed in BC patients, accompanied by a higher ICIs activation. Current studies have shown that the immune system plays an important role in the development and remission of BC, and the main treatment methods for various BC include tumor targeted antibodies (bispecific antibodies), adoptive T cell therapy, vaccines, and immune checkpoint blockade. Personalized immunotherapy remains one of the key factors in improving the prognosis of BC although immunotherapy has made great progress in treating BC. The relationship between MLEM and immune microenvironment not only indicates that immunotherapy has a good prospect in this group, but also suggests that the personalized treatment can be better implemented to improve the prognosis for BC patients based on MLEM.

Chemotherapy is a conventional therapy for treating tumors at present, and the main therapeutic drugs include estrogen antagonists and monoclonal antibody antagonists. The selected therapeutic drugs vary depending on the patient’s conditions. A series of analyses on the sensitivity of BC patients in different groups to chemotherapy were further conducted, and the analyses indicated that patients in high-MLEM group were more suitable for chemotherapy compared to those in low group. Seven potential therapeutic targets and one potential therapeutic drug were identified by the screening of targets and drugs, which may help select personalized treatment plans more suitable for patients with different conditions in clinical practice. Vincristine, a microtubule-disrupting agent, primarily targets mitotic processes; however, recent studies suggest it may influence gene expression indirectly through stress response pathways or modulation of chromatin accessibility (47). While no direct evidence links vincristine to changes in the expression of the nine genes in our model, pathways involving PARP3 and DAXX, both critical in DNA damage response, may be affected by vincristine-induced cellular stress (48, 49). Similarly, other proposed drugs, such as romidepsin (an HDAC inhibitor), have known epigenetic effects that could alter gene expression profiles relevant to breast cancer (50). Future studies, including transcriptomic analyses in treated organoid or *in vivo* models, will be essential to validate these hypotheses.

While the MLEM classifier effectively stratifies patients into high- and low-risk groups with distinct therapeutic sensitivities, experimental validation is critical to confirm these computational predictions. Future work could leverage organoid models derived from patient tumor tissues to test the differential responses of high- and low-MLEM groups to immunotherapy (e.g., PD-1/PD-L1 inhibitors) and chemotherapeutics (e.g., vincristine). Organoids, which replicate the tumor microenvironment, offer a physiologically relevant platform to assess drug efficacy and resistance mechanisms. This approach could also facilitate the development of combination therapies tailored to the molecular profiles of these subgroups, ultimately enhancing the clinical applicability of our findings.

Our study’s strength lies in its robust identification of epigenetic markers and the development of a predictive model. However, the sample size and geographic limitations necessitate further validation across diverse populations to ensure generalizability. Additionally, while the RSF algorithm provides a reliable method for gene selection, the potential biases inherent in machine learning approaches should be acknowledged and addressed in future studies. Our results align with previous research on the significance of epigenetic alterations in cancer prognosis and therapy. Studies have shown that epigenetic modifications can profoundly influence tumor behavior and treatment response. However, our model’s specific focus on BC and the integration of multiple data sources for drug efficacy analysis provide novel insights that enhance its clinical applicability. Future research should aim to validate our findings in larger, more diverse cohorts. Exploring the mechanistic pathways of the identified genes could uncover additional therapeutic targets. Furthermore, integrating genomic, transcriptomic, and epigenomic data could refine the prognostic model, enhancing its precision and utility in personalized medicine.

The MLEM classifier offers a significant advancement in breast cancer prognosis by integrating molecular data into patient stratification, complementing traditional tumor staging. Unlike staging systems that rely on anatomical and pathological features, the MLEM provides molecular insights into tumor biology, enabling more personalized treatment strategies. Low-MLEM patients, characterized by higher immune infiltration, may benefit from immune checkpoint inhibitors such as anti-PD-1/PD-L1 therapies, while high-MLEM patients, less immune-responsive but more sensitive to chemotherapy agents like vincristine, can be managed with tailored chemotherapeutic regimens. Additionally, MLEM could facilitate molecularly stratified clinical trials, improving the precision of therapeutic evaluation. However, several limitations should be acknowledged. The MLEM was developed and validated using retrospective datasets generated from diverse platforms (e.g., HM450K, RNA-seq), which may introduce variability in model performance. Prospective validation in clinical cohorts is essential to confirm its utility. Furthermore, while immunohistochemical validation on clinical samples supports the robustness of the nine-gene signature, additional experimental studies, such as organoid models or *in vivo* experiments, are needed to explore its biological and therapeutic relevance. Finally, the clinical benefit of MLEM compared to established molecular classifiers (e.g., PAM50) warrants further evaluation to solidify its translational potential.

## Conclusion

Nine key epigenetic genes were identified using advanced machine learning techniques, leading to the development of a prognostic model for breast cancer. This model effectively stratified patients into low- and high-MLEM groups, each demonstrating distinct prognostic outcomes and treatment responses. The findings suggest that low-MLEM patients may benefit more from immunotherapy, whereas high-MLEM patients could respond better to chemotherapy, with vincristine showing promise as a therapeutic option. These insights pave the way for more personalized and effective treatment strategies in breast cancer care. Further research is needed to validate these findings and explore the underlying mechanisms.

## Data Availability

The original contributions presented in the study are included in the article/**Supplementary Material**, further inquiries can be directed to the corresponding author.

## References

[1] Coleman MP Quaresma M Berrino F Lutz JM De Angelis R Capocaccia R. Cancer survival in five continents: a worldwide population-based study (CONCORD). Lancet Oncol. (2008) 9:730–56. doi: 10.1016/s1470-2045(08)70179-7 18639491

[2] Polyak K. Heterogeneity in breast cancer. J Clin Invest. (2011) 121:3786–8. doi: 10.1172/jci60534 PMC3195489 21965334

[3] Lehmann BD Bauer JA Chen X Sanders ME Chakravarthy AB Shyr Y. Identification of human triple-negative breast cancer subtypes and preclinical models for selection of targeted therapies. J Clin Invest. (2011) 121:2750–67. doi: 10.1172/jci45014 PMC3127435 21633166

[4] Li Y. Modern epigenetics methods in biological research. Methods. (2021) 187:104–13. doi: 10.1016/j.ymeth.2020.06.022 PMC7785612 32645449

[5] Raghu A Magendhra Rao AKD Rajkumar T Mani S. Prognostic implications of microRNA-155, -133a, -21 and -205 in breast cancer patients' Plasma. Microrna. (2021) 10:206–18. doi: 10.2174/2211536610666210707114843 34238179

[6] Yang S Ji J Wang M Nie J Wang S. Construction of ovarian cancer prognostic model based on the investigation of ferroptosis-related lncRNA. Biomolecules. (2023) 13:306. doi: 10.3390/biom13020306 36830675 PMC9953467

[7] Dawson MA Kouzarides T. Cancer epigenetics: from mechanism to therapy. Cell. (2012) 150:12–27. doi: 10.1016/j.cell.2012.06.013 22770212

[8] Curtis C Shah SP Chin SF Turashvili G Rueda OM Dunning MJ. The genomic and transcriptomic architecture of 2,000 breast tumours reveals novel subgroups. Nature. (2012) 486:346–52. doi: 10.1038/nature10983 PMC3440846 22522925

[9] Marakulina D Vorontsov IE Kulakovskiy IV Lennartsson A Drabløs F Medvedeva YA. EpiFactors 2022: expansion and enhancement of a curated database of human epigenetic factors and complexes. Nucleic Acids Res. (2023) 51:D564–d570. doi: 10.1093/nar/gkac989 36350659 PMC9825597

[10] Liu Z Guo C Dang Q Wang L Liu L Weng S. Integrative analysis from multi-center studies identities a consensus machine learning-derived lncRNA signature for stage II/III colorectal cancer. EBioMedicine. (2022) 75:103750. doi: 10.1016/j.ebiom.2021.103750 34922323 PMC8686027

[11] Wang L Liu Z Liang R Wang W Zhu R Li J. Comprehensive machine-learning survival framework develops a consensus model in large-scale multicenter cohorts for pancreatic cancer. Elife. (2022) 11:e80150. doi: 10.7554/eLife.80150 36282174 PMC9596158

[12] Pal B Chen Y Vaillant F Capaldo BD Joyce R Song X. A single-cell RNA expression atlas of normal, preneoplastic and tumorigenic states in the human breast. EMBO J. (2021) 40:e107333. doi: 10.15252/embj.2020107333 33950524 PMC8167363

[13] McGinnis CS Murrow LM Gartner ZJ. DoubletFinder: doublet detection in single-cell RNA sequencing data using artificial nearest neighbors. Cell Syst. (2019) 8:329–337.e4. doi: 10.1016/j.cels.2019.03.003 30954475 PMC6853612

[14] Suo S Zhu Q Saadatpour A Fei L Guo G Yuan GC. Revealing the critical regulators of cell identity in the mouse cell atlas. Cell Rep. (2018) 25:1436–1445.e3. doi: 10.1016/j.celrep.2018.10.045 30404000 PMC6281296

[15] Baran Y Bercovich A Sebe-Pedros A Lubling Y Giladi A Chomsky E. MetaCell: analysis of single-cell RNA-seq data using K-nn graph partitions. Genome Biol. (2019) 20:206. doi: 10.1186/s13059-019-1812-2 31604482 PMC6790056

[16] Jin S Guerrero-Juarez CF Zhang L Chang I Ramos R Kuan CH. Inference and analysis of cell-cell communication using CellChat. Nat Commun. (2021) 12:1088. doi: 10.1038/s41467-021-21246-9 33597522 PMC7889871

[17] Browaeys R Saelens W Saeys Y. NicheNet: modeling intercellular communication by linking ligands to target genes. Nat Methods. (2020) 17:159–62. doi: 10.1038/s41592-019-0667-5 31819264

[18] Becht E Giraldo NA Lacroix L Buttard B Elarouci N Petitprez F. Estimating the population abundance of tissue-infiltrating immune and stromal cell populations using gene expression. Genome Biol. (2016) 17:218. doi: 10.1186/s13059-016-1070-5 27765066 PMC5073889

[19] Racle J Gfeller D. EPIC: A tool to estimate the proportions of different cell types from bulk gene expression data. Methods Mol Biol (Clifton N.J.). (2020) 2120:233–48. doi: 10.1007/978-1-0716-0327-7_17 32124324

[20] Aran D Hu Z Butte AJ. xCell: digitally portraying the tissue cellular heterogeneity landscape. Genome Biol. (2017) 18:220. doi: 10.1186/s13059-017-1349-1 29141660 PMC5688663

[21] Newman AM Liu CL Green MR Gentles AJ Feng W Xu Y. Robust enumeration of cell subsets from tissue expression profiles. Nat Methods. (2015) 12:453–7. doi: 10.1038/nmeth.3337 PMC4739640 25822800

[22] Finotello F Mayer C Plattner C Laschober G Rieder D Hackl H. Molecular and pharmacological modulators of the tumor immune contexture revealed by deconvolution of RNA-seq data. Genome Med. (2019) 11:34. doi: 10.1186/s13073-019-0638-6 31126321 PMC6534875

[23] Li T Fan J Wang B Traugh N Chen Q Liu JS. TIMER: A web server for comprehensive analysis of tumor-infiltrating immune cells. Cancer Res. (2017) 77:e108–10. doi: 10.1158/0008-5472.Can-17-0307 PMC6042652 29092952

[24] Jiang P Gu S Pan D Fu J Sahu A Hu X. Signatures of T cell dysfunction and exclusion predict cancer immunotherapy response. Nat Med. (2018) 24:1550–8. doi: 10.1038/s41591-018-0136-1 PMC6487502 30127393

[25] Yoshihara K Shahmoradgoli M Martínez E Vegesna R Kim H Torres-Garcia W. Inferring tumour purity and stromal and immune cell admixture from expression data. Nat Commun. (2013) 4:2612. doi: 10.1038/ncomms3612 24113773 PMC3826632

[26] Meyers RM Bryan JG McFarland JM Weir BA Sizemore AE Xu H. Computational correction of copy number effect improves specificity of CRISPR-Cas9 essentiality screens in cancer cells. Nat Genet. (2017) 49:1779–84. doi: 10.1038/ng.3984 PMC5709193 29083409

[27] Yang C Huang X Li Y Chen J Lv Y Dai S. Prognosis and personalized treatment prediction in TP53-mutant hepatocellular carcinoma: an in silico strategy towards precision oncology. Brief Bioinform. (2021) 22:bbaa164. doi: 10.1093/bib/bbaa164 32789496

[28] Wang T Li T Li B Zhao J Li Z Sun M. Immunogenomic landscape in breast cancer reveals immunotherapeutically relevant gene signatures. Front Immunol. (2022) 13:805184. doi: 10.3389/fimmu.2022.805184 35154121 PMC8829007

[29] Wang T Ba X Zhang X Zhang N Wang G Bai B. Nuclear import of PTPN18 inhibits breast cancer metastasis mediated by MVP and importin β2. Cell Death Dis. (2022) 13:720. doi: 10.1038/s41419-022-05167-z 35982039 PMC9388692

[30] Cui P Li H Wang C Liu Y Zhang M Yin Y. UBE2T regulates epithelial-mesenchymal transition through the PI3K-AKT pathway and plays a carcinogenic role in ovarian cancer. J Ovarian Res. (2022) 15:103. doi: 10.1186/s13048-022-01034-9 36088429 PMC9464398

[31] Pan Y Tong JHM Kang W Lung RWM Chak WP Chung LY. EXOSC4 functions as a potential oncogene in development and progression of colorectal cancer. Mol Carcinog. (2018) 57:1780–91. doi: 10.1002/mc.22896 30155936

[32] Azmi AS Bao B Sarkar FH. Exosomes in cancer development, metastasis, and drug resistance: a comprehensive review. Cancer Metastasis Rev. (2013) 32:623–42. doi: 10.1007/s10555-013-9441-9 PMC3843988 23709120

[33] Ding X Zheng D Fan C Liu Z Dong H Lu Y. Genome-wide screen of DNA methylation identifies novel markers in childhood obesity. Gene. (2015) 566:74–83. doi: 10.1016/j.gene.2015.04.032 25871514

[34] Liu J Zhang X Wang H Zuo X Hong L. Comprehensive analysis of purine-metabolism-related gene signature for predicting ovarian cancer prognosis, immune landscape, and potential treatment options. J Pers Med. (2023) 13:776. doi: 10.3390/jpm13050776 37240946 PMC10219580

[35] Beck C Rodriguez-Vargas JM Boehler C Robert I Heyer V Hanini N. PARP3, a new therapeutic target to alter Rictor/mTORC2 signaling and tumor progression in BRCA1-associated cancers. Cell Death Differ. (2019) 26:1615–30. doi: 10.1038/s41418-018-0233-1 PMC6748154 30442946

[36] Rodriguez-Vargas JM Nguekeu-Zebaze L Dantzer F. PARP3 comes to light as a prime target in cancer therapy. Cell Cycle. (2019) 18:1295–301. doi: 10.1080/15384101.2019.1617454 PMC6592235 31095444

[37] Ueda H Akiyama Y Shimada S Mogushi K Serizawa M Matsumura S. Tumor suppressor functions of DAXX through histone H3.3/H3K9me3 pathway in pancreatic NETs. Endocr Relat Cancer. (2018) 25:619–31. doi: 10.1530/erc-17-0328 29599123

[38] Clatterbuck Soper SF Meltzer PS. ATRX/DAXX: guarding the genome against the hazards of ALT. Genes (Basel). (2023) 14:790. doi: 10.3390/genes14040790 37107548 PMC10137841

[39] Wang W Zhou R Wu Y Liu Y Su W Xiong W. PVT1 promotes cancer progression via microRNAs. Front Oncol. (2019) 9:609. doi: 10.3389/fonc.2019.00609 31380270 PMC6644598

[40] Qu H Li X Chen F Zhang M Lu X Gu Y. LncRNA PVT1 influences breast cancer cells glycolysis through sponging miR-145-5p. Genes Genomics. (2023) 45:581–92. doi: 10.1007/s13258-023-01368-8 PMC10113361 36941464

[41] Derderian C Orunmuyi AT Olapade-Olaopa EO Ogunwobi OO. PVT1 signaling is a mediator of cancer progression. Front Oncol. (2019) 9:502. doi: 10.3389/fonc.2019.00502 31249809 PMC6582247

[42] Ma X Li Y Song Y Xu G. Long Noncoding RNA CCDC26 Promotes Thyroid Cancer Malignant Progression via miR-422a/EZH2/Sirt6 Axis. Onco Targets Ther. (2021) 14:3083–94. doi: 10.2147/ott.S282011 PMC8124016 34007185

[43] Duffy MJ O'Grady S Tang M Crown J. MYC as a target for cancer treatment. Cancer Treat Rev. (2021) 94:102154. doi: 10.1016/j.ctrv.2021.102154 33524794

[44] Huang X Qin F Meng Q Dong M. Protein tyrosine phosphatase receptor type D (PTPRD)-mediated signaling pathways for the potential treatment of hepatocellular carcinoma: a narrative review. Ann Transl Med. (2020) 8:1192. doi: 10.21037/atm-20-4733 33241041 PMC7576031

[45] Liu W Huang Y Wang D Han F Chen H Chen J. MPDZ as a novel epigenetic silenced tumor suppressor inhibits growth and progression of lung cancer through the Hippo-YAP pathway. Oncogene. (2021) 40:4468–85. doi: 10.1038/s41388-021-01857-8 34108620

[46] Lv B Wang Y Ma D Cheng W Liu J Yong T. Immunotherapy: reshape the tumor immune microenvironment. Front Immunol. (2022) 13:844142. doi: 10.3389/fimmu.2022.844142 35874717 PMC9299092

[47] Kothari A Hittelman WN Chambers TC. Cell cycle-dependent mechanisms underlie vincristine-induced death of primary acute lymphoblastic leukemia cells. Cancer Res. (2016) 76:3553–61. doi: 10.1158/0008-5472.Can-15-2104 PMC4911277 27197148

[48] Mahmud I Liao D. DAXX in cancer: phenomena, processes, mechanisms and regulation. Nucleic Acids Res. (2019) 47:7734–52. doi: 10.1093/nar/gkz634 PMC6735914 31350900

[49] Boehler C Gauthier LR Mortusewicz O Biard DS Saliou JM Bresson A. Poly(ADP-ribose) polymerase 3 (PARP3), a newcomer in cellular response to DNA damage and mitotic progression. Proc Natl Acad Sci U.S.A. (2011) 108:2783–8. doi: 10.1073/pnas.1016574108 PMC3041075 21270334

[50] Saijo K Imamura J Narita K Oda A Shimodaira H Katoh T. Biochemical, biological and structural properties of romidepsin (FK228) and its analogs as novel HDAC/PI3K dual inhibitors. Cancer Sci. (2015) 106:208–15. doi: 10.1111/cas.12585 PMC4399029 25492515

