# Supplementary material for: Epigenetic profiling for prognostic stratification and personalized therapy in breast cancer
Source: Front Immunol. 2025 Jan 14;15:1510829. doi: 10.3389/fimmu.2024.1510829 (PMC11772270; doi:10.3389/fimmu.2024.1510829)
Supplement: Supplementary file 1 [file DataSheet1.pdf]

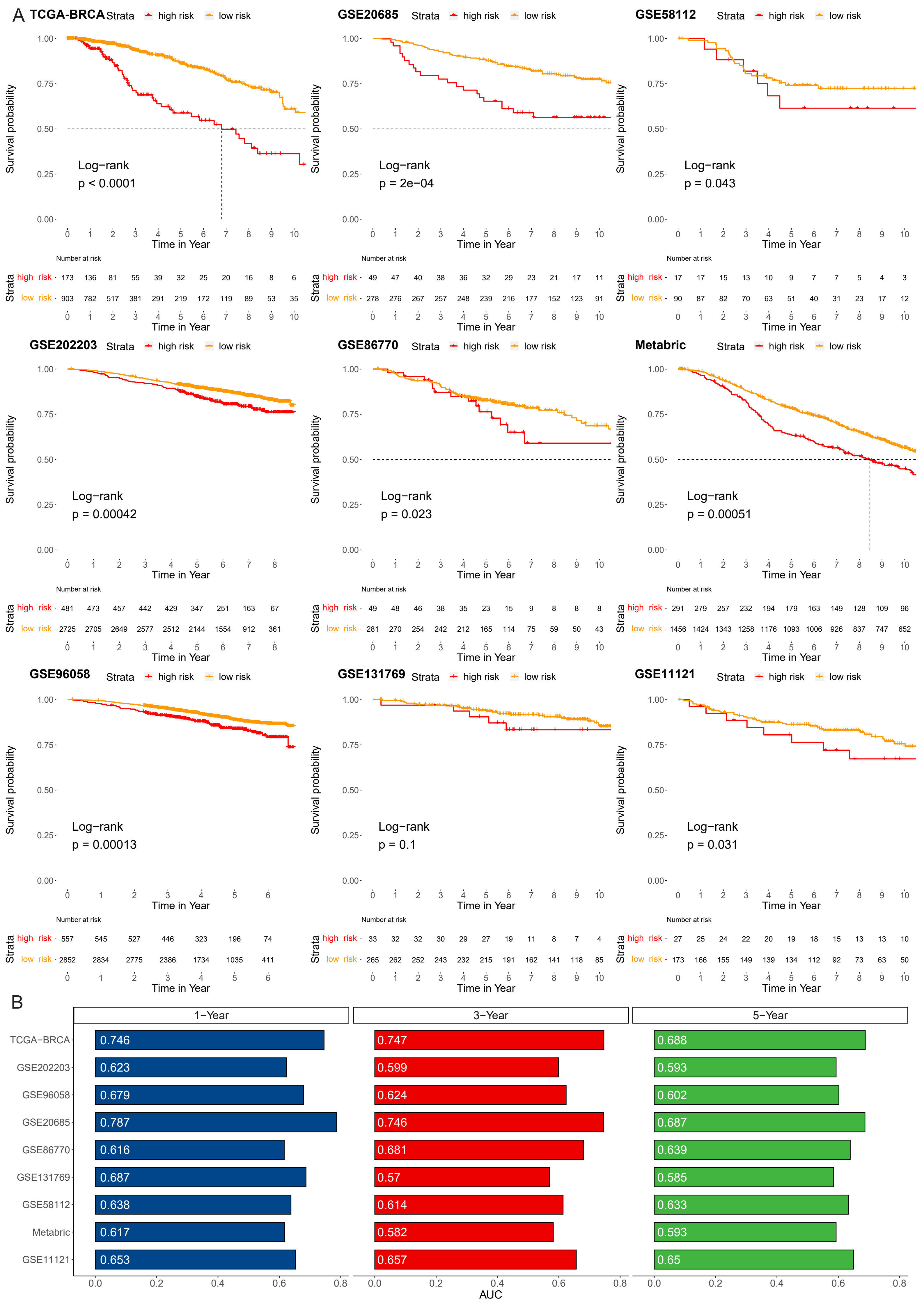

Figure S1. Evaluation of MLEM in 9 cohorts. (A) Kaplan-Meier curves of the MLEM in 9 cohorts. (B) Time-dependent ROC analysis for predicting OS at 1, 3, and 5 years.
