# Supplementary figures and images for: Epigenetic profiling for prognostic stratification and personalized therapy in breast cancer
Source: Front Immunol. 2025 Jan 14;15:1510829. doi: 10.3389/fimmu.2024.1510829 (PMC11772270; doi:10.3389/fimmu.2024.1510829)

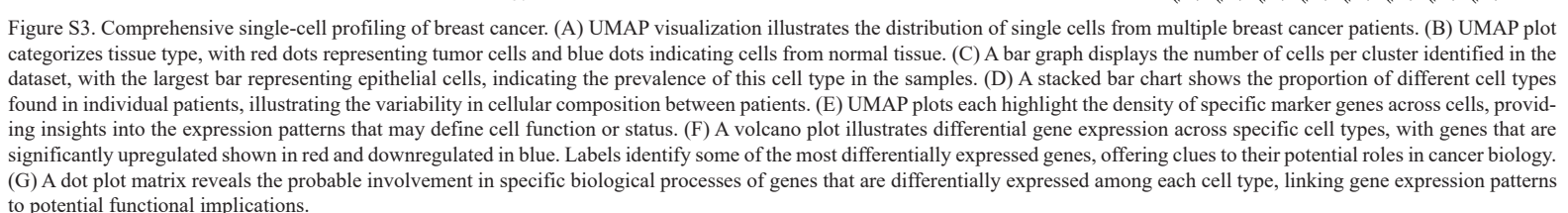

Supplement: Supplementary file 3 [file DataSheet3.pdf]

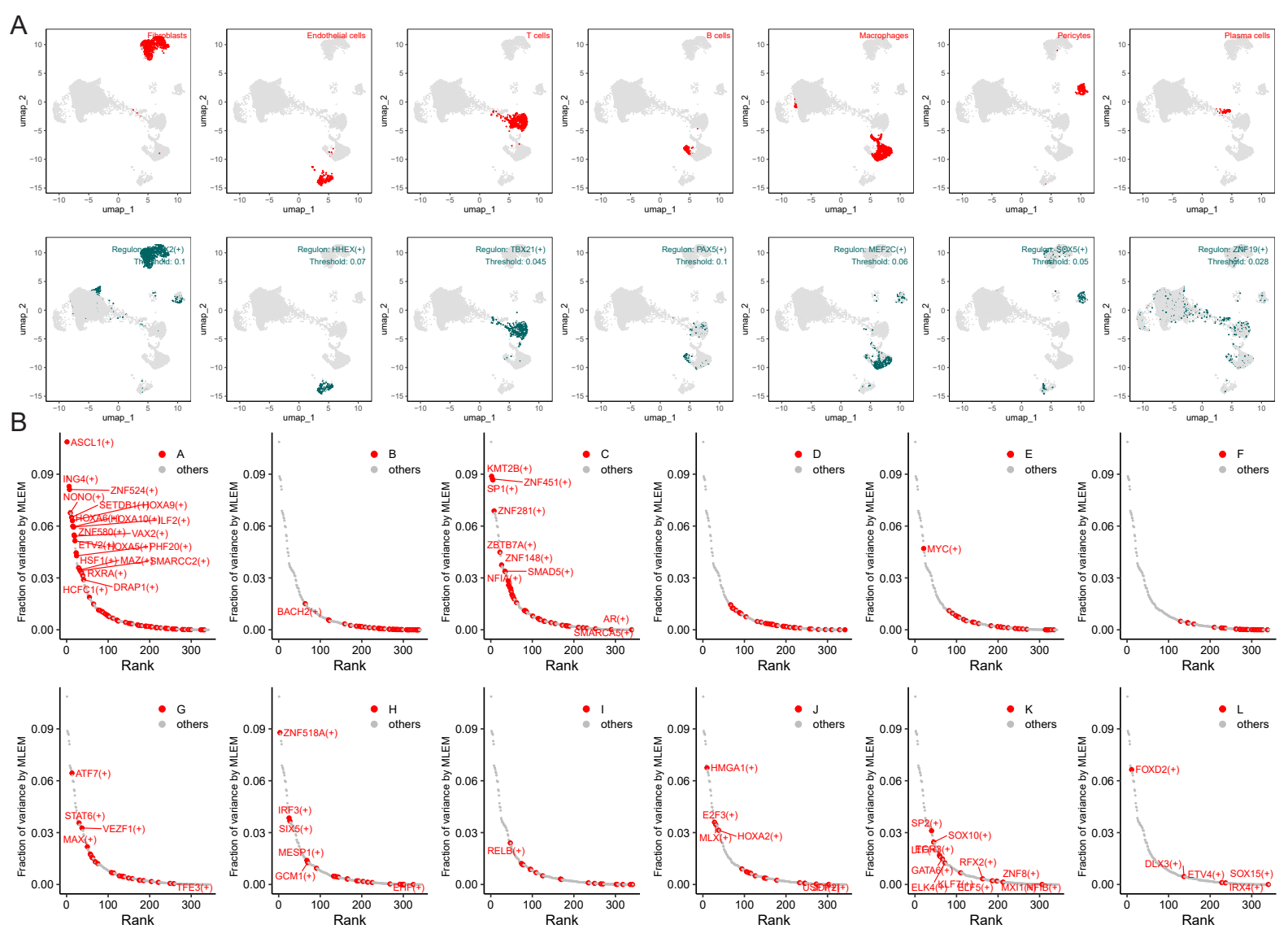

Supplement: Supplementary file 4 [file DataSheet4.pdf]
